# Glycogen Storage Disease Ib and Severe Periodontal Destruction: A Case Report

**DOI:** 10.3390/dj6040053

**Published:** 2018-10-03

**Authors:** Rui Ma, Fardad Moein Vaziri, Gregory J. Sabino, Nima D. Sarmast, Steven M. Zove, Vincent J. Iacono, Julio A. Carrion

**Affiliations:** 1 Private Practice, 1047 Old Post Road, Fairfield, CT 06824, USA; rui.ma.dmd@gmail.com; 2 210-11808 Saint Albert Trail, Edmonton, AB T5L 4G4, Canada; fardadm@gmail.com; 3 Stony Brook University School of Dental Medicine, South Drive, Stony Brook, NY 11794, USA; greg.sabino@gmail.com; 4 Department of Periodontics and Dental Hygiene, The University of Texas School of Dentistry at Houston, 7500 Cambridge Street, Suite 6427, Houston, TX 77054, USA; 5 Department of Periodontology, Stony Brook University School of Dental Medicine, South Drive, Stony Brook, NY 11794, USA; steven.zove@stonybrookmedicine.edu (S.M.Z.); vincent.iacono@stonybrookmedicine.edu (V.J.I.); julio.carrion@stonybrookmedicine.edu (J.A.C.)

**Keywords:** glycogen storage disease, neutrophils, chemotaxis, periodontitis, oral manifestations

## Abstract

**Background**: Glycogen storage diseases (GSDs) are genetic disorders that result from defects in the processing of glycogen synthesis or breakdown within muscles, liver, and other cell types. It also manifests with impaired neutrophil chemotaxis and neutropenic episodes which results in severe destruction of the supporting dental tissues, namely the periodontium. Although GSD Type Ib cannot be cured, associated symptoms and debilitating oral manifestations of the disease can be managed through collaborative medical and dental care where early detection and intervention is of key importance. This objective of the case report was to describe a child with GSD Ib and its associated oral manifestations with microbial, immunological and histological appearances. **Case Presentation**: An eight-year-old Hispanic male with a history of GSD type Ib presented with extensive intraoral generalized inflammation of the gingiva, ulcerations and bleeding, and intraoral radiographic evidence of bone loss. *Tannerella forsythia* was readily identifiable from the biofilm samples. Peripheral blood neutrophils were isolated and a deficient host response was observed by impaired neutrophil migration. Histological evaluation of the soft and hard tissues of the periodontally affected primary teeth showed unaffected dentin and cementum. **Conclusions**: This case illustrates the association between GSD Ib and oral manifestations of the disease. A multi-disciplinary treatment approach was developed in order to establish healthy intraoral conditions for the patient. Review of the literature identified several cases describing GSD and its clinical and radiographic oral manifestations; however, none was identified where also microbial, immunological, and histological appearances were described.

## 1. Introduction

Glycogen storage diseases (GSDs) are genetic disorders that result from defects in the processing of glycogen synthesis or breakdown within muscles, liver, and other cell types [1]. It is estimated to occur in 1 per 20,000 to 25,000 births in the United States. There are at least 10 different types of GSDs known today. GSD I is a rare autosomal recessive disorder that leads to deficiencies of glucose-6-phosphatase catalytic activity (Type Ia) and glucose-6-phosphate translocase (Type Ib) [2]. Clinical manifestations, such as growth retardation [3,4], short stature, doll-like face with fat cheeks, protuberant abdomen and hepatomegaly (due to abnormal glycogen accumulation) [5], inflammatory bowel disease [6], thyroid autoimmunity, and renal disease [7], have been observed and reported in the literature. In addition, patients with GSD Type Ib can also develop neutropenia, as well as impaired neutrophil function, which leads to an increased frequency and severity of bacterial infections [8]. Evidence also suggests that the neutropenia in those with GSD Ib may be caused by increased apoptosis and migration of the neutrophils to inflamed tissues rather than by impairment in maturation [9]. In the oral cavity the neutrophil appears to perform an important role in protecting the periodontal tissues from invasion by pathogenic bacteria resident in the dental biofilm [10].

## 2. Case Presentation

**Figure 1:**
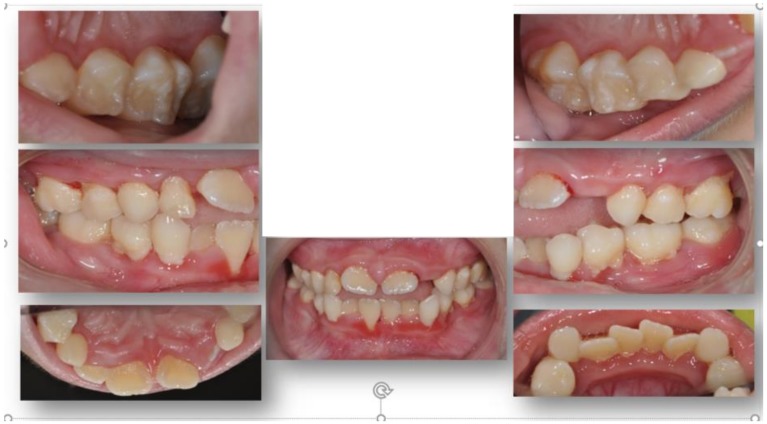
Clinical oral presentation of an eight-year-old male patient with a history of GSD type Ib. Extensive oral inflammation of the supporting periodontal tissues.

**Figure 2:**
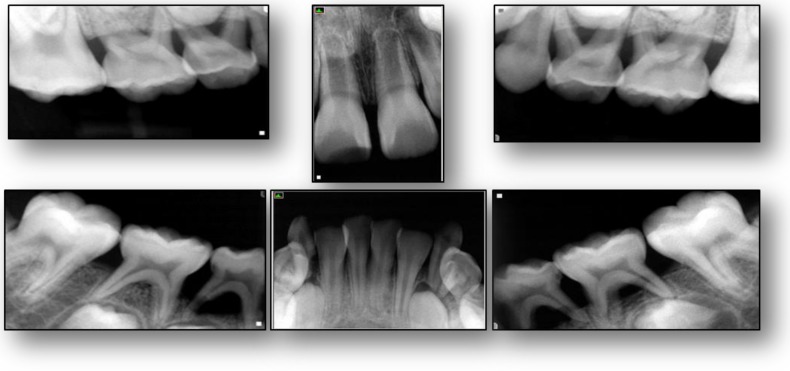
Radiographic presentation of an eight-year-old with a history of GSD type Ib. Radiographic examination reveals severe horizontal bone loss involving the remaining primary dentition.

An eight-year-old Hispanic male presented to the Stony Brook Dental Care Center with a history of GSD type Ib. Oral manifestations of the GSD Ib disease were observed and recorded upon the dental and radiographic examination. Overall, the patient presented with extensive generalized inflammation of the gingiva, erythema, ulceration, and generalized deep periodontal pocketing with bleeding on probing (Figure 1). Generalized severe horizontal bone loss was noted radiographically (Figure 2). Informed consent for treatment was obtained.

**Figure 3:**
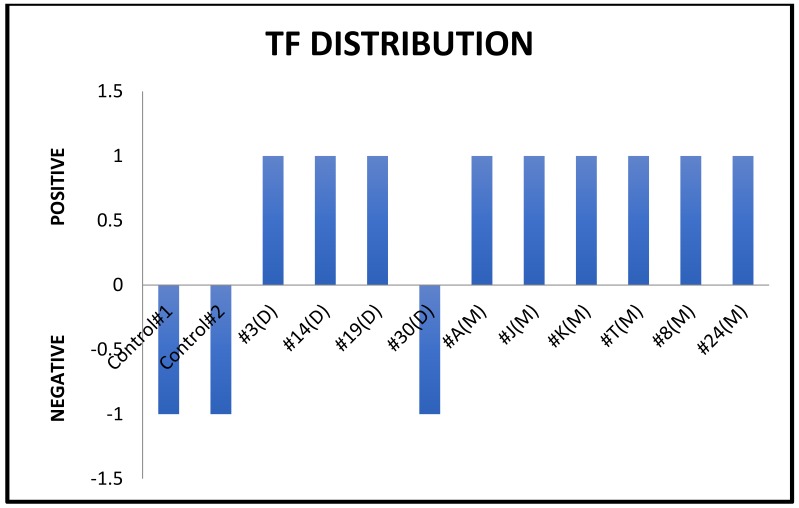
*Tannerella forsythia* distribution by tooth site.

**Figure 4:**
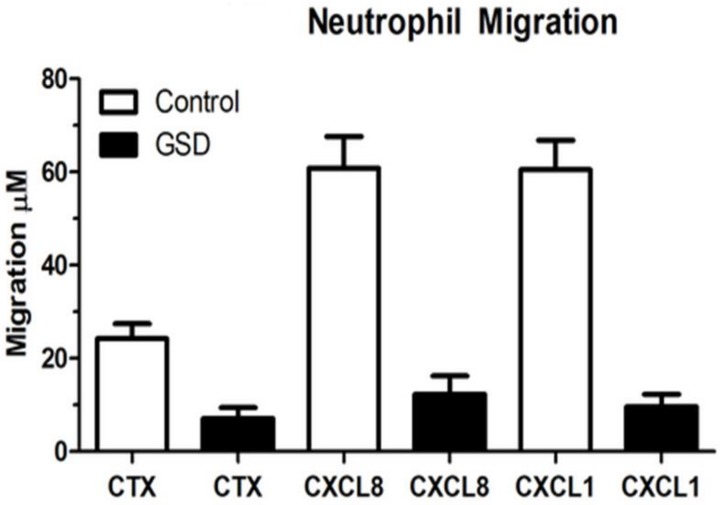
Impaired neutrophil chemotaxis in GSD Ib. Decreased peripheral neutrophil response to the chemokines CXCL8 (IL-8) and CXCL1.

**Figure 5:**
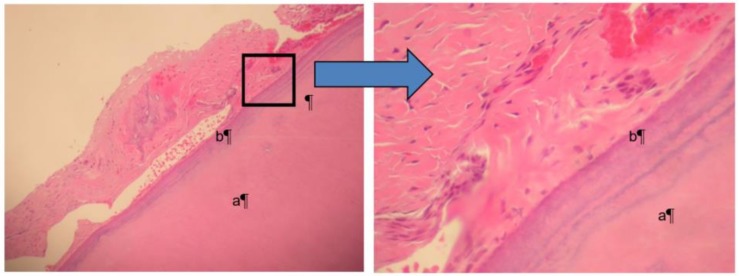
Both pictures show the normal dentin (**a**) surrounded by normal cementum (**b**). The picture on the right magnifies the border between the two structures, which appear to be normal.

Microbial samples were taken with sterile paper points at various primary and permanent teeth to demonstrate the periodontal pathogen distribution [11]. A blood sample was drawn in order to study systemic neutrophil migration. Peripheral blood neutrophils were isolated according to a standard protocol [12] and suspended in HBSS + 10 mM HEPES (pH 7.4) and 1% BSA. A 48-well Boyden chamber apparatus (Neuro Probe, Inc., Gaithersburg, MD, USA) was arranged so that 20 nM of CXCL1 (R&D Systems, Minneapolis, MN, USA), 20 nM of CXCL8 (R&D Systems), or HBSS + 10 mM HEPES (pH 7.4) and 1% BSA was added as the chemoattractant or control in the bottom portion of the chamber. A 5-μm 35 cellulose nitrate filter (Neuro Probe, Gaithersburg, MD, USA) was placed between the two halves of the Boyden chamber. Neutrophils in a volume of 50 μL, at no more than 4 × 10^6^ cells/mL, were loaded into the top chamber and allowed to migrate for 15 min at 37 °C. The filter was fixed in 100% 2-propanol, stained with Harris-type hematoxylin, clarified with xylene, and mounted for analysis. The distance that neutrophils traveled into the filter was measured using the leading-front method via bright-field microscopy. The microbial composition of the oral biofilm was characterized by multiplex PCR. 16S rRNA gene was used as the primers in PCR. Sterilized deionized water was used as negative control. Of the common putative periodontal pathogens, *Tannerella forsythia* was readily identifiable from the biofilm samples (Table 1, Figure 3). In addition, a deficient host response was observed by impaired neutrophil migration in response to the chemokines CXCL1 and CXCL8 (Figure 4). Histological evaluation [13] of the soft and hard tissues of the periodontally affected primary teeth showed a normal attachment apparatus, including bone, cementum, and periodontal ligament (Figure 5).

Based on the clinical findings and the understanding of the disease, a treatment plan was developed collaboratively with the Departments of Orthodontics, Pediatric Dentistry, and Periodontology. All remaining primary teeth had a hopeless prognosis and it was elected to proceed with extractions after obtaining informed consent. No postoperative infections or bleeding were reported or observed. In order to preserve the space for the remaining succedaneous teeth, a nance appliance and lower lingual holding arch were fabricated for the maxillary and mandibular dentitions, respectively. A two to three month recall interval for dental examinations and preventative care has been recommended for this patient [14]. Patient was not followed up in this case report after immediate post-operative treatment course.

## 3. Discussion

Current available evidence indicates that the neutrophil serves a protective role in the periodontium [15,16,17,18]. Thus, individuals with aberrant neutrophil production or behavior often have early-onset, severe forms of gingivitis and/or periodontitis [19,20]. This is particularly evident in patients whose neutrophils are chemotactically defective. In this case report, two chemokines were used to measure neutrophil migration. CXCL1 is a small cytokine, which is secreted by human melanoma cells and expressed by macrophages, neutrophils, and epithelial cells [21,22]. Study has shown that it is critical for neutrophil-dependent bacterial elimination via induction of reactive oxygen species [23]. CXCL8, also called interleukin 8, which is also a neutrophil chemotactic factor and is produced by macrophages as well as other cells types. Both chemokines are responsible to induce chemotaxis and attract neutrophils to migrate toward the site of infection. The patient with GSD type Ib in this report had defective neutrophil chemotaxis in response to the chemokines CXCL1 and CXCL8 in comparison to normal neutrophils. In addition, PCR analysis indicated the presence of the “Red Complex” microorganism (which includes *Porphyromonas gingivalis*, *Tannerella forsythia*, and *Treponema denticola* [24]), *Tannerella forsythia*, which was a major periodontal pathogen in conjunction with a compromised host immune response that was responsible for severe periodontal attachment destruction in this eight-year-old patient. Conversely, no histological cemental, and dentinal abnormalities were detected. Although GSD Type Ib cannot be cured, the disease and associated symptoms can be managed through comprehensive medical and dental care. In this case report, the decision of removing all the remaining primary teeth was based on the severe localized horizontal bone loss. These areas have the most plaque accumulation, clinically, as well. Due the mobility of the primary teeth, patient was not comfortable to eat in the area. Nance appliance and lingual holding were placed in order to minimally maintain the edentulous space and prevent posterior teeth from shifting mesially. In patients with GSD Type Ib, dental care should be focused on primary prevention and early recognition of dental and periodontal diseases. Understanding the pathophysiology of GSD Ib will enhance the ability for its clinical management and, hopefully, for the future development of a cure.

## Figures and Tables

**Table 1:** Periodontal pathogen distribution table by tooth site.

	Sample Site	AA	PG	TD	TF
**1**	Control#1	-	-	-	-
**2**	Control#2	-	-	-	-
**3**	#3(D)	-	-	-	+
**4**	#14(D)	-	-	-	+
**5**	#19(D)	-	-	-	+
**6**	#30(D)	-	-	-	-
**7**	#A(M)	-	-	-	+
**8**	#J(M)	-	-	-	+
**9**	#K(M)	-	-	-	+
**10**	#T(M)	-	-	-	+
**11**	#8(M)	-	-	-	+
**12**	#24(M)	-	-	-	+

AA = *Aggregatibacter actinomycetemcomitans*; PG = *Porphyromonas gingivalis*; TD = *Treponema denticola*; TF = *Tannerella forsythia*; “+” represents corresponding periodontal pathogen is present; “-” represents corresponding periodontal pathogen is absent.
